# Giant coronary artery aneurysms: a case series and management recommendations

**DOI:** 10.1093/ehjcr/ytaf561

**Published:** 2025-11-10

**Authors:** Arja Suzanne Vink, Maik J D Grundeken, Bart Straver, Danielle Robbers-Visser, Berto J Bouma, Dave R Koolbergen, Robbert J De Winter, Marcel A M Beijk

**Affiliations:** Department of Clinical and Experimental Cardiology, Amsterdam UMC, Location University of Amsterdam, Room G2-207, PO Box 22660, Amsterdam 1100 DD, The Netherlands; Department of Cardiology, Center for Congenital Heart Disease Amsterdam-Leiden (CAHAL), Room G2-207, PO Box 22660, Amsterdam 1100 DD, The Netherlands; Department of Clinical and Experimental Cardiology, Amsterdam UMC, Location University of Amsterdam, Room G2-207, PO Box 22660, Amsterdam 1100 DD, The Netherlands; Department of Cardiology, Center for Congenital Heart Disease Amsterdam-Leiden (CAHAL), Room G2-207, PO Box 22660, Amsterdam 1100 DD, The Netherlands; Department of Cardiology, Center for Congenital Heart Disease Amsterdam-Leiden (CAHAL), Room G2-207, PO Box 22660, Amsterdam 1100 DD, The Netherlands; Department of Pediatrics, Division of Pediatric Cardiology, Emma Children’s Hospital, Amsterdam UMC, Room H8-240, PO Box 22660, Amsterdam 1100 DD, The Netherlands; Department of Clinical and Experimental Cardiology, Amsterdam UMC, Location University of Amsterdam, Room G2-207, PO Box 22660, Amsterdam 1100 DD, The Netherlands; Department of Cardiology, Center for Congenital Heart Disease Amsterdam-Leiden (CAHAL), Room G2-207, PO Box 22660, Amsterdam 1100 DD, The Netherlands; Department of Clinical and Experimental Cardiology, Amsterdam UMC, Location University of Amsterdam, Room G2-207, PO Box 22660, Amsterdam 1100 DD, The Netherlands; Department of Cardiology, Center for Congenital Heart Disease Amsterdam-Leiden (CAHAL), Room G2-207, PO Box 22660, Amsterdam 1100 DD, The Netherlands; Department of Cardiothoracic Surgery, Center for Congenital Heart Disease Amsterdam-Leiden (CAHAL), Leiden University Medical Center, Leiden 2333 ZG, The Netherlands; Department of Cardiothoracic Surgery, Amsterdam UMC, Location University of Amsterdam, Room G2-207, PO Box 22660, Amsterdam 1100 DD, The Netherlands; Department of Clinical and Experimental Cardiology, Amsterdam UMC, Location University of Amsterdam, Room G2-207, PO Box 22660, Amsterdam 1100 DD, The Netherlands; Department of Cardiology, Center for Congenital Heart Disease Amsterdam-Leiden (CAHAL), Room G2-207, PO Box 22660, Amsterdam 1100 DD, The Netherlands; Department of Clinical and Experimental Cardiology, Amsterdam UMC, Location University of Amsterdam, Room G2-207, PO Box 22660, Amsterdam 1100 DD, The Netherlands; Department of Cardiology, Center for Congenital Heart Disease Amsterdam-Leiden (CAHAL), Room G2-207, PO Box 22660, Amsterdam 1100 DD, The Netherlands

**Keywords:** Coronary artery aneurysm (CAA), Coronary artery ectasia (CAE), Percutaneous coronary intervention (PCI), Coronary artery bypass grafting, Case report

## Abstract

**Background:**

Coronary artery aneurysms (CAAs) are a rare clinical entity, and little is known about their natural history, prognosis, and management. Invasive management of patients with CAA is challenging not only because of this limited understanding but also due to the technical challenges associated with both percutaneous and surgical interventions.

**Case summary:**

We describe the management of seven cases with giant CAAs in an elective setting. Our first case demonstrates successful percutaneous closure of a giant right coronary aneurysm in an asymptomatic patient using an Amplatzer™ Vascular Plug. In four cases, surgery was performed, due to the presence of symptoms and/or when revascularization by a percutaneous intervention was considered to be unsuitable. Two cases were managed conservatively due to the patients’ advanced age and significant comorbidities.

**Discussion:**

Although most published cases of giant CAAs involve elective surgical treatment, growing expertise shows that percutaneous intervention is a viable and increasingly promising alternative—particularly in younger patients or those at high surgical risk. Based on the limited available evidence and our own experience, we propose a practical management algorithm to help guide multidisciplinary decision-making. This emphasizes that percutaneous treatment should be actively considered as part of a tailored, patient-specific strategy in the management of giant CAAs.

Learning points

- Coronary artery aneurysms (CAAs) are a rare clinical entity. Little is known about their natural history and prognosis, making the decision for invasive management challenging.
- Although most case reports on patients with giant CAAs describe surgical treatment in an elective setting, there is increasing experience with percutaneous interventions, making it a promising technique in patients who are young or at high surgical risk.
- Invasive treatment strategies for giant CAAs should be individualized based on the clinical presentation, aetiology, coronary anatomy, and morphology of CAA. Considering this patient-tailored approach, a multidisciplinary heart team is indispensable in the decision-making.

## Introduction

A coronary artery aneurysm (CAA) is a focal dilatation of a coronary segment of at least 1.5 times the adjacent normal segment.^1,2^ It is a rare anatomical disorder of the coronary arteries with a reported incidence of <1.0%.^3^ The pathogenesis and mechanisms of development of CAAs are not well understood; however, the underlying aetiology appears to vary with age. Congenital anomalies and inflammatory or connective tissue disorders are more commonly seen in younger individuals, whereas in older patients, atherosclerotic degeneration—with chronic inflammation and medial destruction—is typically the predominant mechanism.^4^ Coronary artery aneurysms have been associated with poor long-term outcomes irrespective of the presence of concomitant atherosclerotic coronary artery disease (CAD).^5,6^ Clinical presentation ranges from incidental findings on cardiac imaging to acute coronary syndrome (ACS).^4^ Coronary artery aneurysm can manifest clinically as a consequence of (i) concomitant obstructive atherosclerotic disease; (ii) local thrombosis leading to distal embolization and myocardial infarction; (iii) compression of adjacent structures due to massive enlargement of the CAA; (iv) rupture of the CAA; and (v) stress-induced myocardial ischaemia due to microvascular dysfunction.^3^

Due to the poorly elucidated underlying mechanisms,^3^ the variable presentation, and the lack of large-scale outcome data on invasive treatment modalities, CAAs pose a challenge to the managing clinician. We describe the management of seven cases with giant CAAs (>20 mm or dilatation exceeding the reference vessel diameter by >4 times), in an elective setting (*Table 1*), followed by a review of the current literature, and propose an algorithm for the management of patients with CAA.

**Table 1:** Patient characteristics

Case	Sex	Age	Presentation	Symptoms	Physical examination	Comorbidities	Maximum size and location of CAA	Complications	Intervention	Follow-up (years)
**1**	Male	64	Screening	No	Unremarkable	No	38 × 38 mm and 78 × 85 mm (RCA)	Asymptomatic inferoseptal-inferior myocardial infarction 2 years after diagnosis	Amplatzer™ Vascular Plug II into proximal RCA, 9 years after diagnosis due to significant enlargement	11
**2**	Female	69	Incidental finding	Yes, dyspnea 1 year after diagnosis	Unremarkable	No	33 × 36 mm (RCA), 57 × 66 mm (Cx)	Fistulas to the coronary sinus with a dilated LV (LVED 59 mm) with moderate MV and TV regurgitation and SPAP of 58 mmHg	Surgery with fistula closure and complete aneurysm exclusion, 1 year after diagnosis due to the size of the aneurysms and presence of symptoms	two
**3**	Female	44	Medical screening	No	Unremarkable	Anomalous left coronary artery from the pulmonary artery (ALCAPA), AF	75 × 95 mm (RCA), 21 mm (LAD), 21 mm (Cx)	None	At diagnosis re-implantation LCA in aorta. Ten years later, exclusion and resection RCA aneurysm + CABG (Ao-PDA) due to the size of the aneurysm and a good patency of the PDA	15
**4**	Male	12	M. Kawasaki	Yes, angina pectoris	Unremarkable	No	4.5 × 4.5 mm, 22 × 22 mm, and 15 × 15 mm (RCA), 15 × 15 mm (LAD)	None	CABG (LIMA-LAD, (Y)RIMA-PDA) at the age of 22 years after treatment with warfarin and aspirin due to angina pectoris and ischaemia on MRI	26
**5**	Male	31	M. Kawasaki	Yes, NSTEMI	Unremarkable	No	81 × 88 mm (RCA), 14 × 15 mm (Cx), 15 × 29 mm (LAD)	Ambulant inferolateral myocardial infarction	Resection of the aneurysm in the RCA and CABG (LIMA-LAD –(Y)RIMA-MO-PDA) at age 46 years due to significant enlargement of the aneurysms at a presentation with NSTEMI	16
**6**	Male	64	During follow-up after CABG (LIM_A-LAD, Ao-D1-D2, Ao-MO-RPL-RDP), and re-CABG (Ao-LAD, Ao-RDP)	No	Unremarkable	Severe mitral valve regurgitation	37 × 43 mm and 52 × 61 mm (Cx)	None	MitraClip implantation 1 year later as a consequence of endocarditis from a mechanical mitral valve implantation (mini sternotomy), CAA was left conservative due to comorbidity	8
**7**	Male	76	Incidental finding	Yes, NSTEMI and possible rupture	Unremarkable	No	19 × 19 mm (RCA), 16 × 19 mm (Cx), 30 × 37 mm (LAD)	NSTEMI 1 year after diagnosis and 3 years after diagnosis possible rupture with cardiac tamponade during excessive anticoagulation	Pericardial puncture and correction of anticoagulant, afterwards stabilization. Aneurysms were not grown in size and were therefore left conservative	3

## Summary figure

**Figure:**
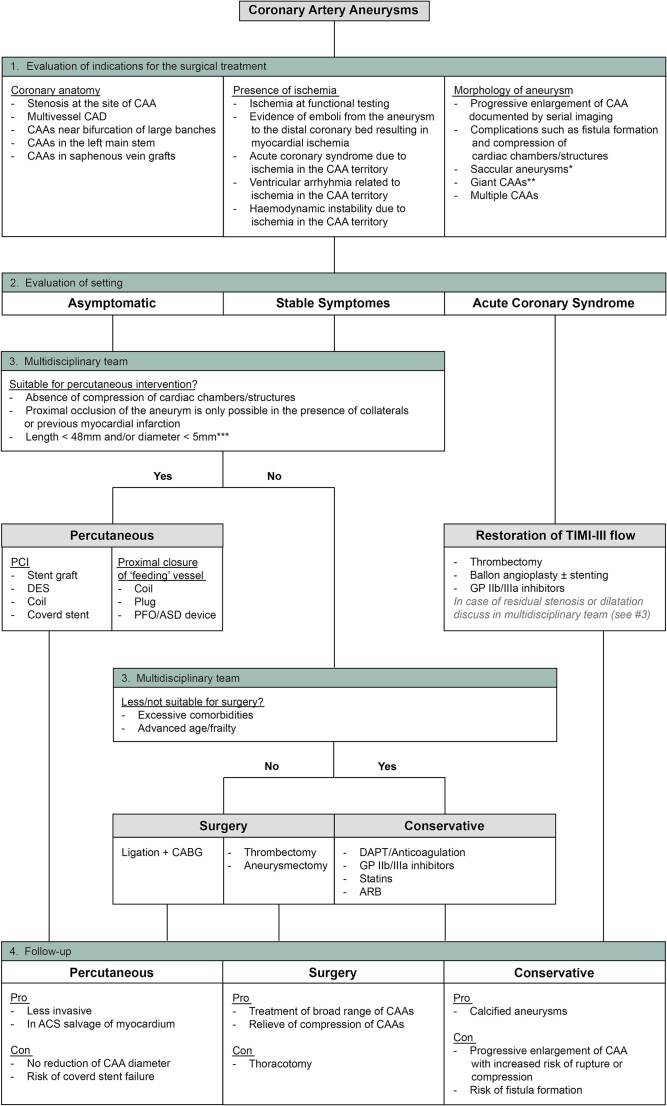
A suggested algorithm for the management of patients with coronary artery aneurysm. *Saccular aneurysms are more prone to thrombosis and rupture^3,7^. **A size of >20 mm or dilatation exceeding the reference vessel diameter by >4 times is considered a giant aneurysm.^3^. ***Aneurysms with a small length can be treated with a single covered stent. As the re-endothelialization of covered stents is very slow, a drug eluting stent (DES) can be placed inside the covered stent with a longer size. For aneurysms with a long length, a long DES can be implanted first whereafter covered stents can be placed inside.

## Case presentation

### Patient 1: percutaneous intervention

In 2012, an asymptomatic 64-year-old male was referred to our cardiology clinic after a routine transthoracic echocardiogram detected an abnormal structure between the right atrium and right ventricle adjacent to the tricuspid valve. An additional, cardiac magnetic resonance imaging (cMRI) and coronary angiography were performed, revealing an aneurysm of 34 × 39 mm in size at the mid-segment of the right coronary artery (RCA) with a large thrombus inside. The patient was treated with vitamin K antagonists and followed up at our outpatient clinic. Two years later, his electrocardiogram showed new Q waves in the inferior leads, without having experienced any symptoms. A cMRI was repeated showing an inferoseptal-inferior myocardial infarction without viability of the myocardium. In addition, a second aneurysm with a size of 18 × 18 mm was visualized slightly proximal from the previously known aneurysm, which itself was not increased in size but still had a large thrombus. A P2Y12 inhibitor was added to the treatment with vitamin K antagonists.

**Figure 1:**
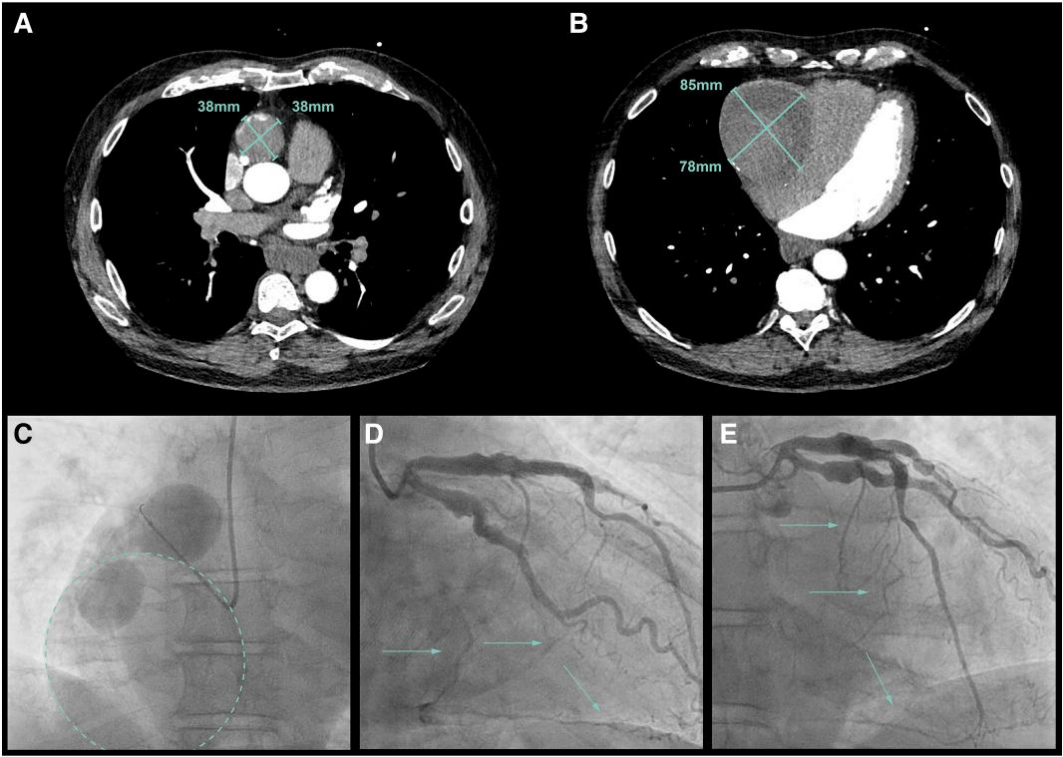
(*A*) A 38 × 38 mm aneurysm of the proximal segment of the right coronary artery. (*B*) A 78 × 85 mm aneurysm in the mid-right coronary artery. (*C*) Slow filling of the proximal aneurysm of the right coronary artery and part of the aneurysm in the mid-right coronary artery. The dashed line indicates the aneurysm in the mid-right coronary artery of 78 × 85 mm that is thrombosed and calcified. The distal right coronary artery is occluded. (*D*) Dilated proximal LAD and circumflex artery with epicardial collaterals from the septal branches and the obtuse marginal artery to the distal part of the right coronary artery (arrows). (*E*) Amplatzer™ Vascular Plug inserted into the proximal right coronary artery.

During follow-up, biennial cardiac computed tomography (CCT) showed progressive enlargement of the aneurysms over time reaching 38 × 38 mm proximally and 78 × 85 mm in the mid-RCA by 2021 (*Figure 1A* and *B*). Distal from the aneurysm in the mid-segment of the RCA, the RCA was occluded with collaterals coming from the left coronary artery (LCA) (*Figure 1C–E*). The patient was still asymptomatic; however, due to the significant enlargement of the aneurysms, the risk of rupture was considered high. The patient was discussed in the multidisciplinary heart team. As the patient was asymptomatic, and the distal RCA was occluded with collaterals from the LCA, proximal RCA occlusion by percutaneous intervention was preferred over coronary artery bypass grafting (CABG) to close the ‘feeding’ vessel. A percutaneous femoral procedure was then performed using a 6 French catheter (CLS 3.5). An *Amplatzer*™ *Vascular Plug* (AVP; AVP2-008 mm) was inserted into the proximal RCA under fluoroscopic guidance (Supplementary material online, *Case 1 Video 1a* and *b*). After placement of the AVP, there were no cardiac symptoms, and the electrocardiogram showed no significant changes.

Today, the patient is still followed up at our outpatient clinic and remains asymptomatic with a good exercise tolerance. As expected, his last transthoracic echocardiogram in 2023 showed that the largest aneurysm remained stable without any growth.

### Patients 2, 3, 4, and 5: surgical intervention

**Figure 2:**
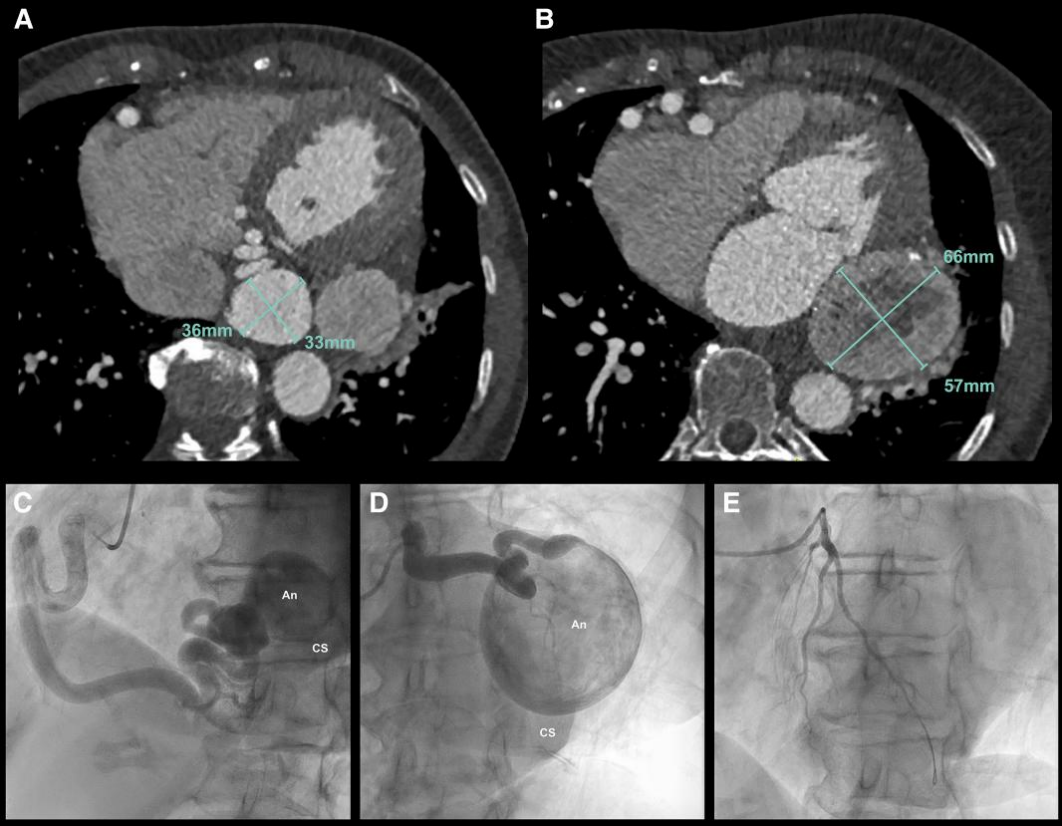
(*A*) Dilated right coronary artery with distal a 33 × 36 mm aneurysm with a connection to the coronary sinus. (*B*) Dilated circumflex artery with after the obtuse marginal artery a 57 × 66 mm aneurysm. Two vessels appear to emerge from the aneurysm, both of which drain into the coronary sinus. (*C*) Dominant dilated right coronary artery. From the right coronary artery with an aneurysm of the posterolateral branch, a spherical aneurysm is shown draining into the coronary sinus. (*D*) Dilated circumflex artery with a fistula from the obtuse marginal artery to a spherical aneurysm draining into the coronary sinus. (*E*) Normal diameter of the LAD.

In our case series, four patients underwent surgery for correction of their CAA. Patient 2, a 69-year-old female, was referred after an incidental finding during trauma screening following a 2020 cycling accident. Computed tomography and an additional CCT revealed an aneurysm of the RCA (33 × 36 mm) and circumflex artery (Cx) (57 × 66 mm) with suggestions for a fistula to the coronary sinus (CS) (*Figure 2A* and *B*). Coronary angiography (*Figure 2C* and *D*, Supplementary material online, *Case 2 Video 2a-d*) showed a dilated RCA with an aneurysm of the posterolateral (RPL) branch and a fistula to the CS. The Cx was dilated with a fistula from the obtuse marginal artery to an aneurysm draining into the CS. Due to the size of the aneurysms and the risk of rupture, closure of the CAAs was indicated. A percutaneous intervention was considered unsuitable due to the size of the CAAs and the presence of fistulas. When the patient developed exertional in 2021, surgery was performed to exclude the aneurysms and close the coronary fistulas. Postoperatively, she became asymptomatic, and a 6-month CCT showed no new aneurysms and patent coronary arteries without fistulas.

**Figure 3:**
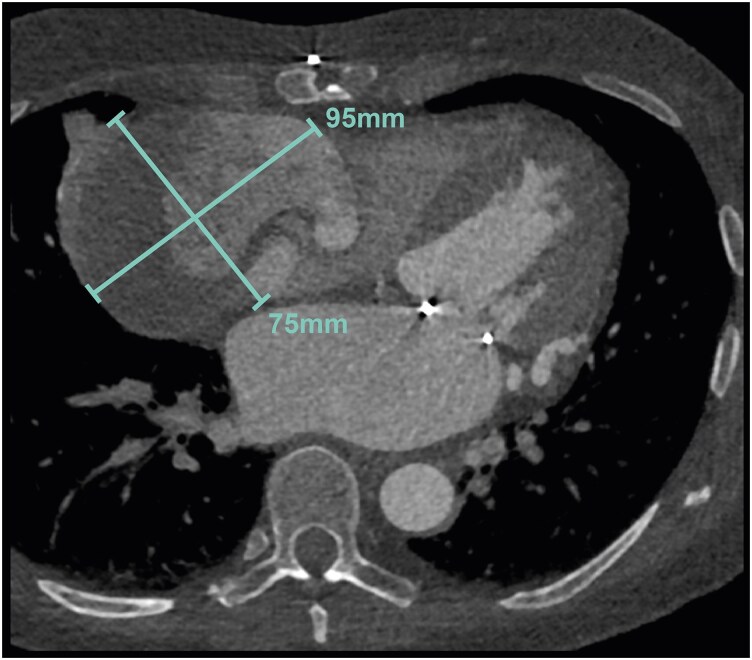
Cardiac computed tomography imaging of Patient 3 showing a dilated right coronary artery with a thrombosed 75 × 95 mm aneurysm.

Patient 3, a 44-year-old female, presented with atrial fibrillation and underwent CT scanning for CAD assessment, revealing an anomalous LCA from the pulmonary artery (ALCAPA or Bland–White–Garland syndrome). At that time, the coronary arteries were dilated (origo left main 10 mm, left anterior descending artery (LAD) 12 mm, and origo RCA 10 mm) with two aneurysms in the RCA from 32 and 26 mm. She underwent surgical re-implantation of the LCA in the aorta and mitral valve repair for moderate regurgitation caused by left atrial dilatation from atrial fibrillation. Ten years later, RCA aneurysms enlarged to 75 × 95 mm (*Figure 3*). Due to the large size of the aneurysms, occluding the RCA was the only percutaneous option. Because the posterior descending artery (PDA) remained patent, surgical aneurysm exclusion and resection combined with CABG using a venous graft to the PDA was preferred to avoid inducing an inferior myocardial infarction. Currently, 4 years later, she remains asymptomatic though CT revealed a new 26 mm aneurysm in the Cx.

**Figure 4:**
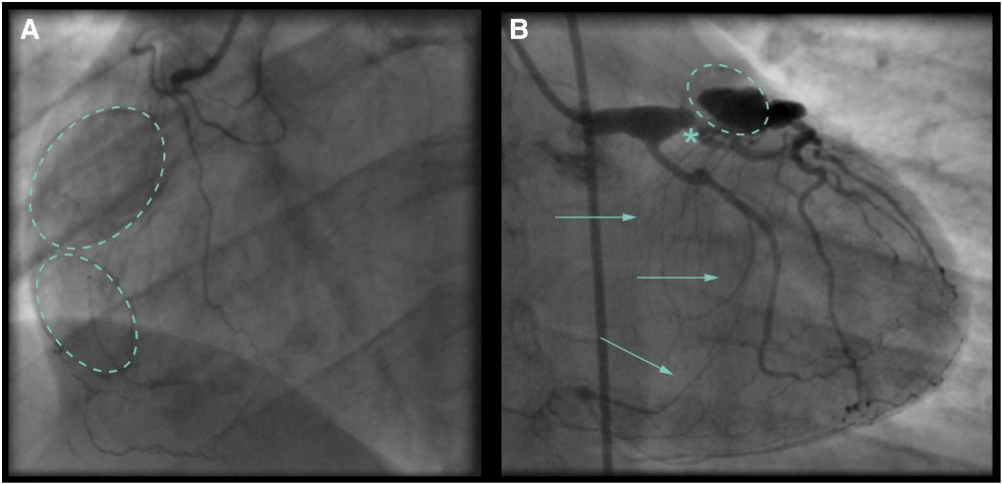
(*A*) Proximal occluded right coronary artery with two large thrombosed and calcified aneurysms (dashed line). (*B*) Significant stenosis of the proximal LAD (*) with a smaller partly thrombosed and calcified aneurysm (dashed line). The arrows show epicardial collaterals to the right coronary artery (Supplementary material online, *Case 4 Video 4a-c*).

**Figure 5:**
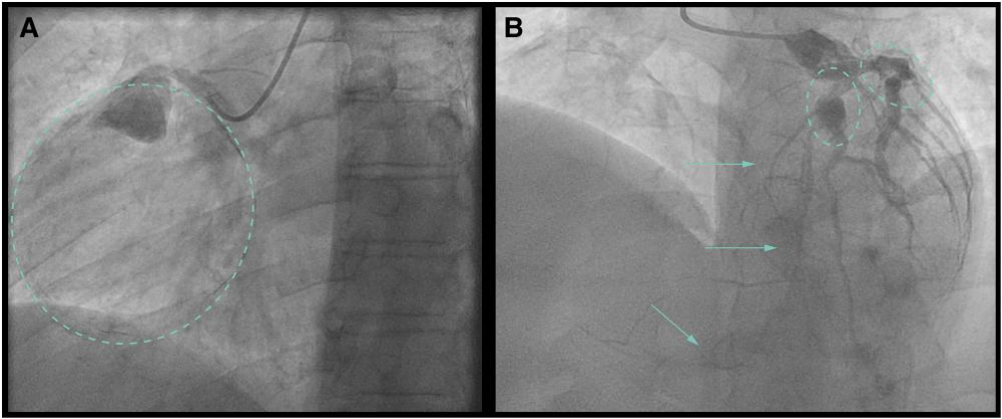
(*A*) Proximal occluded right coronary artery with a large thrombosed and calcified aneurysm (dashed line). (*B*) Two partly thrombosed and calcified aneurysms in the LAD and ramus circumflexus (dashed lines). The arrows show epicardial collaterals to the right coronary artery (Supplementary material online, *Case 5 Video 5a-c*).

Both Patients 4 and 5 (*Figures 4* and *5*) had Morbus Kawasaki at a young age. Both patients had excessive growth of their CAAs during follow-up and became symptomatic. Therefore, they both underwent surgery with CABG.

### Patients 6 and 7: conservative

Patient 6 underwent CABG at age 45 and re-CABG at age 53 due to graft occlusion. At 64, he presented with angina pectoris. Coronary angiography showed patent grafts and a proximal Cx aneurysm of 28 × 37 mm. During follow-up, he developed a severe mitral valve regurgitation due to posterior leaflet retraction. At age 70, he was treated with a MitraClip™. During that procedure, transoesophageal echocardiogram revealed a large thrombus with spontaneous contrast in the Cx aneurysm. An additional CT scan showed two giant aneurysms in the Cx of 37 × 43 mm and 52 × 61 mm. As these were asymptomatic, no intervention was performed. Six months later, the patient unfortunately developed mitral valve endocarditis with recurrent severe mitral valve regurgitation. He underwent mitral valve replacement surgery, but the Cx aneurysms were left untreated due to the complexity of the operation. At age 72, the patient died from metastatic pancreatic carcinoma.

Patient 7 had a CT scan at the age of 76 years for screening, revealing aneurysms in the RCA (19 × 19 mm), Cx (16 × 19 mm), and LAD (30 × 37 mm). One year later, he had a non-ST-elevation myocardial infarction that was treated conservatively. At age 79, he presented in cardiogenic shock due to cardiac tamponade. During pericardiocentesis, 750 cc haemorrhagic fluid was aspirated. After correction of a supratherapeutic International Normalized Ratio, he stabilized, and there was no recurrence of pericardial fluid. The aneurysms were treated conservatively.

## Discussion

### Case overview

We describe seven cases of giant CAAs not presenting with ACS, each managed with a different approach. Our first case demonstrates a safe and successful percutaneous closure of a giant native right coronary aneurysm in an asymptomatic patient using an AVP. In four cases, surgery was performed, due to the presence of symptoms and/or because percutaneous revascularization was deemed unsuitable. Two cases were treated conservatively, due to advanced age and significant comorbidities.

### Current evidence on coronary artery aneurysm management

Most studies on CAA management focus on patients presenting with ACS. Only a few case reports and small case series have described the outcome of percutaneous intervention in asymptomatic patients,^8,9^ or those with angina or dyspnoea.^3,10^ The same scarcity applies to the surgical treatment of CAAs.^10^ Furthermore, studies directly comparing percutaneous coronary intervention (PCI) and surgery in CAA patients are lacking. However, cohort studies (regardless of clinical presentation) report no major differences in important cardiovascular outcomes between PCI and surgery.^5,6^

### Challenges in decision-making for non-acute coronary syndrome patients

Given the limited evidence on invasive treatment of CAA in non-ACS patients, treatment decisions can be complex. If an intervention is considered—due to certain high-risk clinical or anatomical features, the choice of treatment modality should be tailored to coronary anatomy, presence of ischaemia, and/or morphology of the aneurysm.

### Surgical vs. percutaneous management

In patients with stable angina or no symptoms, surgery is most often preferred, especially in the presence of multiple or giant CAAs (>20 mm or dilatation exceeding the reference vessel diameter by >4 times), fistula, or compression of cardiac structures.^3,11^ In selected cases, a percutaneous intervention can be preferred to either (i) exclude the aneurysm using stents, coils, or covered stents, or (ii) occlude the ‘feeding’ vessel to prevent further enlargement of the CAA using coils, plugs, or closure devices (either patent foramen ovale or atrial septal defect devices). Closure of the ‘feeding’ vessel can be considered when collaterals are present such as in our first case presentation. Although coronary fistulas in the context of giant CAAs are extremely rare and are usually treated surgically, percutaneous closure has been performed in several cases.^12–14^ Importantly, percutaneous closure does not reduce the size of the aneurysm.

### Conservative management

Patients unsuitable for surgery and percutaneous intervention—due to anatomy, frailty, or comorbidities—may be managed conservatively with pharmacotherapy alone.

### Towards a practical algorithm

Given the rarity of CAAs and the limited evidence available, managing these patients remains highly complex and challenging. It is precisely because of this scarcity of data that consolidating clinical experience into a practical management algorithm is crucial. Such a guide can provide valuable support and structure to multidisciplinary teams faced with difficult treatment decisions.

Based on our experience and the limited literature, we propose a modified management algorithm that builds on earlier frameworks^3,15,16^ (see *Summary Figure*). A key contribution of our work is the more detailed guidance on when a percutaneous approach may be considered, as well as clarification of the intended therapeutic goal when selecting a specific percutaneous technique. By linking the anatomical and clinical context to the rationale behind each technique, we aim to promote a more goal-directed and individualized approach to percutaneous treatment.

In addition, we offer a comparative overview of the advantages and limitations of all available management strategies—surgical, percutaneous, and conservative—to aid clinicians in selecting the most appropriate option for each patient. This algorithm is designed to support structured, patient-centred, and evidence-informed decision-making within the multidisciplinary team.

### Limitations

Given the rarity of giant CAAs, this case series is limited by its small sample size, potential case selection bias, and lack of a control group. The absence of direct comparisons between treatment strategies also limits conclusions about relative efficacy. Additionally, the findings may not be generalizable to the broader CAA population. Nonetheless, the series offers relevant clinical insights into this rare condition.

## Conclusion

Invasive treatment of CAAs should be individualized based on clinical presentation, aetiology, coronary anatomy, and aneurysm morphology. A patient-tailored approach is essential, and multidisciplinary team discussion remains a cornerstone of optimal management.

## Lead author biography


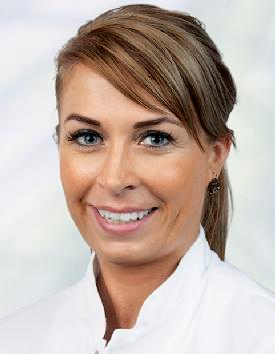


Arja Suzanne Vink is a cardiologist at Amsterdam UMC and an interventional cardiology fellow. Her special interests include coronary physiology and congenital heart disease.

## Supplementary Material

ytaf561_Supplementary_Data

## Data Availability

The data underlying this article cannot be shared publicly due to the privacy of individuals that participated in the study. The data will be shared on reasonable request to the corresponding author.
